# Effectiveness of motor control exercise, aerobic walking, and muscle strengthening programs in improving outcomes in a subgroup of population with chronic low back pain positive for central sensitization: a study protocol for a randomized controlled trial

**DOI:** 10.1186/s13063-023-07316-x

**Published:** 2023-05-09

**Authors:** G. Shankar Ganesh, Abdur Raheem Khan, Sakti Prasad Das, Ashfaque Khan, Raee S. Alqhtani, Adel Alshahrani, Mohammad Abdulrehman Mohammad Jarrar, Hashim Ahmed

**Affiliations:** 1 Composite Regional Centre for Skill Development, Rehabilitation, and Empowerment of Persons with Disabilities, Lucknow, Uttar Pradesh 226017 India; 2 Present Address: Department of Physiotherapy, Integral University, Kursi Road, Lucknow, Uttar Pradesh 226026 India; 3 Swami Vivekanand National Institute of Rehabilitation Training and Research, Cuttack Dt, Odisha 754010 India; 4 Department of Medical Rehabilitation Sciences- Physiotherapy Program, College of Applied Medical Sciences, Najran University, Najran, 55461 Kingdom of Saudi Arabia

**Keywords:** Central nervous system sensitization, Chronic pain, Executive function, Exercise, Low back pain, Quality of life, Randomized clinical trial

## Abstract

**Background:**

The role of pain sensitivity in the development and maintenance of chronic pain states, impaired executive functioning, and patient recovery is being investigated. Conditioned pain modulation (CPM) is widely used to measure musculoskeletal pain associated with central sensitization (CS). Despite the recommendations of many reviews and clinical practice guidelines that exercise programs reduce pain and disability, the overall confidence in these results is considered “critically low.” The “active ingredient” of exercise programs and the dominant factor influencing CPM remain largely unknown.

The objectives of this trial are to determine:

• If different exercises cause different results on the CPM in a subgroup of people with chronic low back pain (CLBP) who are labeled as having CS pain,

• If a program of exercise interventions for 12 weeks would alter executive functioning, quality of life (QoL), disability, and pain in persons with CLBP.

• The relationship between patient characteristics, executive functions, CPM, and QoL

**Methods:**

The trial is a randomized, controlled, multi-center study with four experimental groups and one healthy control group. Both the researchers and the people in the study will be blinded to the results. This paper describes the protocol for a trial examining the effects of 12-week individualized, twice-weekly exercise sessions lasting 30 to 60 min in persons with CLBP, who are positive for CS. Participants will be randomized to receive either patient education with motor control exercises (MCE), superficial strengthening (SS), aerobic exercises (AE), or patient education alone. Another group comprised of healthy volunteers will serve as controls. The primary outcomes are changes in CPM outcomes as measured by the cold pressor test (CPT). The secondary objectives are to evaluate executive functioning, pain, disability, quality of life, and spine muscle strength. The outcomes will be measured at 3 months and at a 6-month follow-up.

**Discussion:**

The outcomes of the study will help in gaining more information and evidence about exercise-induced analgesia from the perspective of CPM. Measuring exercise outcomes will aid in scientifically prescribing exercise prescriptions in people with CLBP. The study outcomes will also assist in identifying the characteristics of individuals who will respond or respond indifferently to exercises. Investigating the relationship between the study’s various outcomes could provide information for future trials.

**Trial registration:**

Clinical Trials Registry of India (CTRI) identifier: CTRI/2022/03/041143. Registered on 16 March 2022.

**Supplementary Information:**

The online version contains supplementary material available at 10.1186/s13063-023-07316-x.

## Administrative information

**Table:** 

Title {1}	Effectiveness of motor control exercise, aerobic walking, and muscle strengthening programs in improving outcomes in a subgroup of population with chronic low back pain positive for central sensitization: a study protocol for a randomized controlled trial
Trial registration {2a and 2b}	Clinical Trials Registry of India (CTRI) identifier: CTRI/2022/03/041143
Protocol version {3}	Issue Date: October 15, 2022 Protocol Amendment Number: 03
Funding {4}	The Deanship of Scientific Research, Najran University, Kingdom of Saudi Arabia, funds the article processing fee
Author details {5a}	Conceptualization: SG Methodology: SG, ARK, SD,AK Data Collection: SG, ARK, AK Formal analysis and investigation: SG, ARK, SD,AK,RSA,AA,MM, HA Writing – SG Writing—review and editing: All authors read and approved the final manuscript
Name and contact information for the trial sponsor {5b}	Investigator initiated clinical trial; Shankar Ganesh (Principal investigator) shankarpt@rediffmail.com
Role of sponsor {5c}	This is research initiated by the investigator. The funders played no role in the study design and in the collection, analysis, and interpretation of data and in the writing of the manuscript
Roles of committees {5d}	The Research Committee, comprised of the Dean, Doctoral Studies, Dean, Research & Development, Dean, Physiotherapy, Head of the Department of Physiotherapy, one internal and one external expert, and one supervisor, will oversee the trial and review the progress of the study to facilitate the smooth running of the study. The medical superintendent and clinical supervisor will be responsible for overseeing participant safety, including serious unexpected suspected adverse events and database integrity, respectively

## Introduction

### Background and rationale {6a}

Chronic low back pain (CLBP) refers to low back pain (LBP) that is persistent and incapacitating for more than 3 months [1] and is unrelated to underlying illnesses such as an infection, tumor, or fracture [2]. Because the causes of most CLBP conditions are unknown, they are categorized as non-specific CLBP (NSCLBP) [3]. Studies have indicated abnormal cortical function in CLBP patients including reduced left dorsolateral prefrontal cortex activity [4]; disruption of executive functions such as multitasking ability [5], sustained attention and working memory [6, 7]; longer processing time [8]; and substantial disturbances in mental flexibility, delayed memory, and psychomotor speed [9].

The role of pain sensitivity in the development and maintenance of chronic pain states and its influence on patient recovery are unclear and thus being investigated. Impaired functioning of descending anti-nociceptive mechanisms and activation of the descending and ascending pain facilitatory pathways facilitate overall pain transmission. Central sensitization (CS) is the increased responsiveness of nociceptive neurons in the central nervous system (CNS) to their normal or subthreshold afferent input [10]. Conditioned pain modulation (CPM) is extensively used to measure CS-associated musculoskeletal pain and refers to the quantitative sensory test used to assess the functionality of endogenous pain inhibition in the CNS [11]. CPM is directly related to the cognitive performance and self-reported health-related quality of life (QoL) [12].

Although persons with chronic pain are believed to have lower CPM inhibition, Sluka (2016) reported that some individuals with fibromyalgia and healthy persons exhibit normal and abnormal responses to CPM, respectively [13]. Evidence for CPM alterations in CLBP patients is mixed, with studies revealing impaired [14], normal [15], and facilitated [16] CPM compared with healthy controls. Mlekusch et al. (2016) evaluated endogenous pain modulation by using the cold pressor test (CPT) in persons with and without LBP and reported that the CPM effect declined more rapidly in the LBP group than in the normal population [17].

Exercises reduce the overall sensitivity of the CNS [18] by activating exercise-induced endogenous analgesia and spinal inhibitory mechanisms. Some studies have concluded that a bout of aerobic exercise (AE) could reduce sensitivity to painful stimuli in healthy individuals; however, other studies have reported contradictory results, with AE producing both hyper and hypoalgesic responses [19]. A study revealed an increase and a decrease in mean pain thresholds, respectively, in patients with CLBP and chronic fatigue syndrome as a response to exercise (submaximal AE program on a bicycle ergometer) [20]. Vaegter et al. (2016) examined CPM and exercise-induced hypoalgesia (EIH) in patients with chronic musculoskeletal pain under two different training conditions (bicycling and isometric contraction): one with high pain sensitivity and the other with low pain sensitivity. A partial impairment of CPM and EIH was observed in patients with high pain sensitivity compared with that in patients with low pain sensitivity [21]. Past reviews and guidelines have barely provided sufficient details about the indications of different exercise types to be used in clinical practice.

Few studies have investigated how different exercise doses affect EIH in chronic pain patients. Other studies have compared the EIH response following an AE condition with a predefined intensity (such as 75% of the maximal heart rate) to that with a self-selected intensity [22, 23]. Furthermore, numerous studies on EIH in chronic pain populations have relied on exercise doses that might not accurately reflect clinical practice, such as AE protocols lasting for < 15 min [22–24] and isometric exercises at low loads (10–30% of the maximum voluntary contraction) instead of dynamic resistance exercises [25, 26].

No previous study has investigated the associations between CPM outcomes, executive functions, and QoL in CLBP patients from the perspective of exercise interventions. Additional studies investigating the effects of varied exercise types and dosage on EIH in chronic pain populations that more closely resemble those observed in clinical practice are warranted. Furthermore, knowledge about the ideal frequency and types of exercise to be recommended in an exercise prescription is limited. We postulated that studying the effect of three types of exercise interventions in a subgroup of CS-positive patients and exploring the relationship between CPM responses, CLBP-related outcomes, and executive functions would increase our understanding regarding exercise prescriptions. This is crucial because pain sensitization-targeting interventions are limited and evidence for the effectiveness of exercise interventions is inconclusive.

### Objectives {7}

The primary objective of the present study is to determine if different exercise types produce varied outputs on CPM in a subgroup of CLBP patients classified as having CS pain. The secondary objectives are to evaluate if a 12-week exercise intervention program would alter executive functioning, QoL, disability, and pain and to examine the relationship between patient characteristics, executive functions, CPM, and QoL in CLBP patients.

### Trial design {8}

**Fig. 1:**
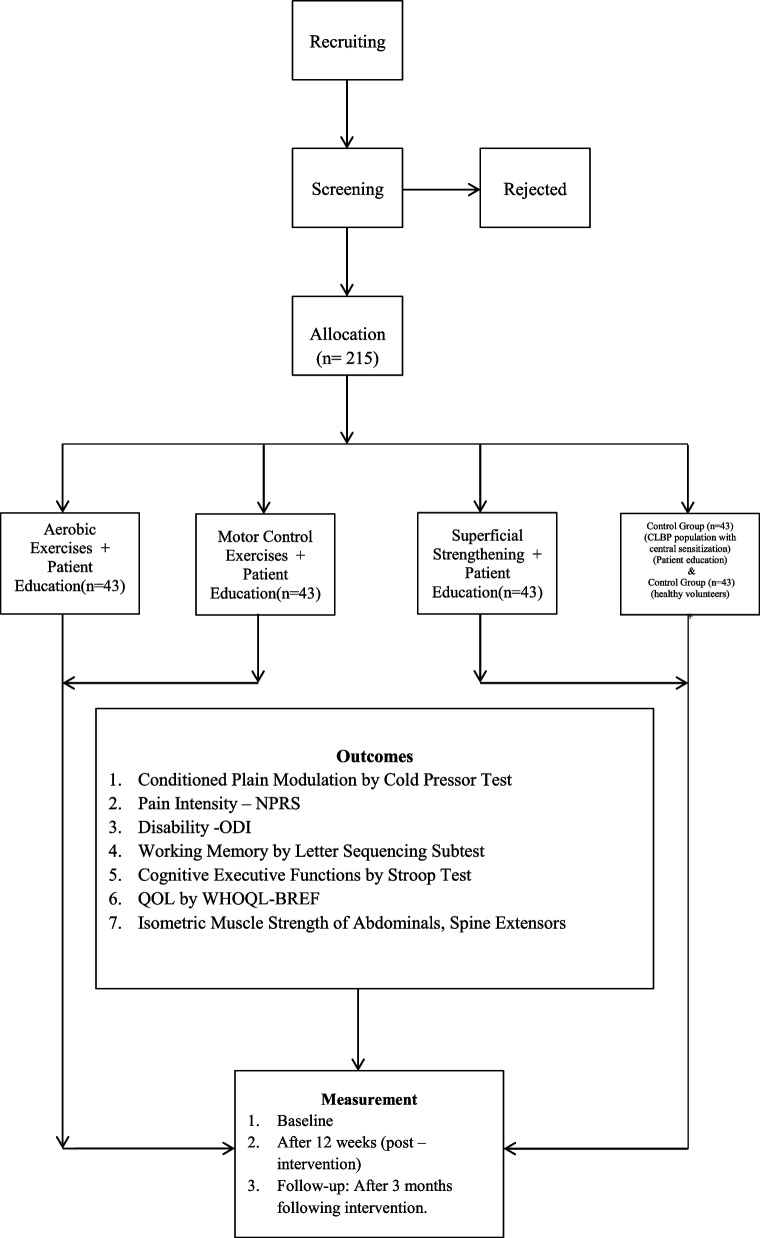
Schematic of study design

This is a randomized, controlled, double-blinded study determining how CPM changes in response to three types of active exercises in CS-positive CLBP patients. Participants will be randomized to undergo 12 weeks of the motor control exercise (MCE), superficial strengthening (SS), or AE combined with patient education or patient education alone. This active treatment concurrent control design will be used to demonstrate superiority of one exercise group over another. All patients will be allocated in a 1:1:1:1 ratio using random number tables. All participants will receive advice to stay active. Another no-pain control group will also be recruited. The intervention will be provided for 12 weeks. An independent assessor will collect data at baseline (t0), after intervention at 3 months (t1), and at the 6-month follow-up (t2). Outcome measures include CPT for measuring CPM, letter–number sequencing subtest in the Wechsler Adult Intelligence Scale-III Edition (WAIS-III), Stroop neurophysiological test, QoL assessed using the abbreviated WHOQOL-BREF questionnaire, isometric muscle strength of spine and hip muscles, the Oswestry Disability Index (ODI) questionnaire, and the Numerical Pain Rating Scale (NPRS). Table 1 and Fig. 1 present the study design and flowchart, respectively. The research ethics approval was granted by the Integral University, India (IIAHSR/DO/PT/2022/23), and Composite Regional Centre for Skill Development, Rehabilitation, and Empowerment of Persons with Disabilities, India (CRCL/Ph.D. Data Collection/2021–22/1556). The study is designed according to the Consolidated Standards of Reporting Trials (CONSORT) guidelines and reported according to the Standard Protocol Items: Recommendations for Interventional Trials (SPIRIT) checklist. The study will use a 5 (between subjects − type of exercise interventions: MCE vs. AE vs. SS vs. patient education vs. no-pain control) × 3 (within subjects − time: before vs. after vs. 3 months after the intervention) mixed design.

**Table 1:**
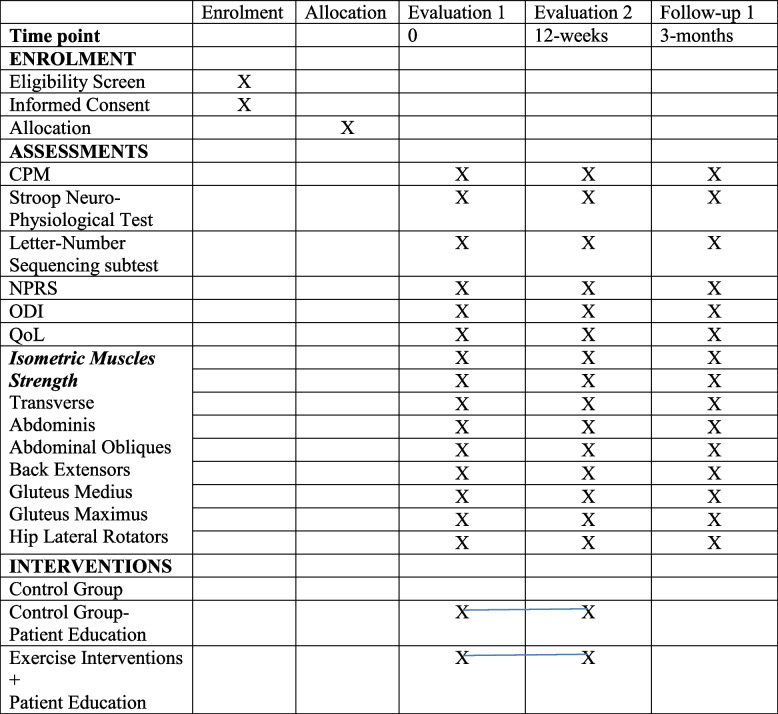
Schedule of enrolment, intervention and assessment

*CPM* Conditioned pain modulation, *NPRS* Numerical Pain Rating Scale, *ODI* Oswestry Disability Index, *QoL* Quality of life

## Methods: participants, interventions, and outcomes

### Study setting {9}

The study will be conducted between August 2022 and October 2023. In total, 172 participants (both male and female) aged between 18 and 59 years who have CLBP and are positive for CS-related signs and symptoms will be recruited from the Outpatient Physiotherapy Departments of the Integral University and the Composite Regional Centre for Skill Development, Rehabilitation, and Empowerment of Persons with Disabilities. Similarly, we will recruit a no-pain control population (*n* = 43).

### Eligibility criteria {10}

The inclusion criteria for the participants are as follows: age between 18 and 59 years, male or female sex, diagnosed with NSCLBP satisfying the definition provided in the European guidelines for NSCLBP management [27], and being positive for CS-related signs and symptoms [28]. Further, using the Physical Activity Readiness Questionnaire, all participants will be screened for contraindications to exercise [29]. Table 2 presents the complete inclusion and exclusion criteria.

**Table 2:** Inclusion and exclusion criteria

Inclusion criteria	Exclusion criteria
• Persons between 18 and 59 years of age • CLBP of more than 3 months duration • Pain intensity of more than 4 on the NPRS • Clear mechanical basis of the symptoms • Positive for CS-related sign and symptoms • Cognitively intact [Mini-Mental State Examination score > 24] • Basic working knowledge of English language	• Attributable to a recognizable, known specific pathology (e.g., infection, tumor, osteoporosis, fracture, structural deformity, inflammatory disorder (e.g., ankylosing spondylitis) or cauda equina syndrome) • Participants who are contra-indicated to participate in physical activity • Participants who have undertaken any surgical procedures to spine or suffering from symptomatic spine diseases • Participants, who report acute pain within 48 h prior to the appointment • No corticosteroids or other drug therapy in the preceding 2 weeks • Participants suffering from any other chronic pain condition, high blood pressure, Raynaud’s disease, frostbite, any open cut, sore, or bone fracture, other major illness such as cancer, visual and/or hearing impairment, and psychiatric disorders • Presence of clinical signs or symptoms of radiculopathy

### Who will take informed consent? {26a}

Patients will be introduced to the trial by the research team. This team will answer any questions during an in-person meeting. The primary investigator will provide information sheets about the proposed trial to all participants. The research team members will discuss the trial with the participants in light of the information provided in the information sheets. The patient will then be able to engage in an informed discussion with the primary investigator. The primary investigator will obtain the signed written consent from the patients interested in participating in the trial. The consent form will include details such as the study’s title, investigators’ names, registered information, research background, how the study will be conducted, what participants are expected to do in the study, treatment plans, and obligations. The information sheets and patient consent forms will be translated into Hindi and shared with the participants if they do not understand English.

### Interventions

#### Explanation for the choice of comparators {6b}

The control group populations comprising the no-pain population and CS-positive patients with CLBP will help determine the effectiveness of interventions and allow us to gather valuable information for a better understanding of the impact of endogenous analgesia on pain control. The choice of a comparator is further strengthened by the results of recent recommendations that clinicians should employ standard education strategies, including exercise and physical activity, for CLBP patients [30].

#### Intervention description {11a}

All patients who report for CLBP management will be continuously enrolled in the study. They will undergo a standardized history taking for recording of their demographic details and will be screened for eligibility. The first author will conduct a complete physical assessment and neurological examination, and the presence of “red flags” will be closely monitored. The patients providing voluntary informed consent will be referred to the outpatient departments (OPDs) of the concerned study center where more detailed evaluations will be conducted.

In case of any doubt about a participant’s capacity to engage in the exercise program, they will be directed to a medical professional for an evaluation. The same process will continue until all participants have been recruited (*n* = 172). The recruited participants will then be randomized to receive one of the three exercise interventions along with patient education or patient education alone. A group comprising healthy volunteers will serve as the control group. The interventions will last for 12 weeks. Follow-up will be conducted 3 and 6 months after the intervention.

Three qualified physiotherapists (PTs) in each center will provide the interventions. All of them have a postgraduate degree in musculoskeletal physiotherapy and an average of 11 years of clinical experience. Before initiation of the study, they will attend a training session discussing the detailed treatment procedures. All PTs will be instructed to adhere to the treatment checklist provided and to complete treatment notes after each session. The participants will be instructed to avoid engaging in any other physical program during the study period and to not exercise at home during the intervention period. All the three participant groups will receive the targeted exercise interventions along with the same health education provided to group 4 participants. In order to manage time as effectively as possible, every exercise group will have 5–7 participants. The participants will be advised to continue the exercises after the treatments are over in order to support self-management.

Group 1 (AE group): This group will be subjected to a comprehensive stretching routine (lower limbs, upper limbs, and spine for 30 s in each section), 20–40 min of treadmill walking (intensity adjusted based on individual capacity and pain reports), and a 5-min local massage to relax [31]. Each walking session will start with a 5-min warm-up at a self-determined speed, followed by 20–40 min of intensive walking, and finally, a 5-min cool-down at a self-selected speed. The intensity of the walking exercise will be determined based on the heart rate and using the Borg scale. The maximum heart rate % will be calculated as 208 − (0.7 × age) [32]. The levels between 12 and 13 (moderate intensity) of the Borg scale and a range of 50–75% of maximum heart rate will be used.

**Fig. 2:**
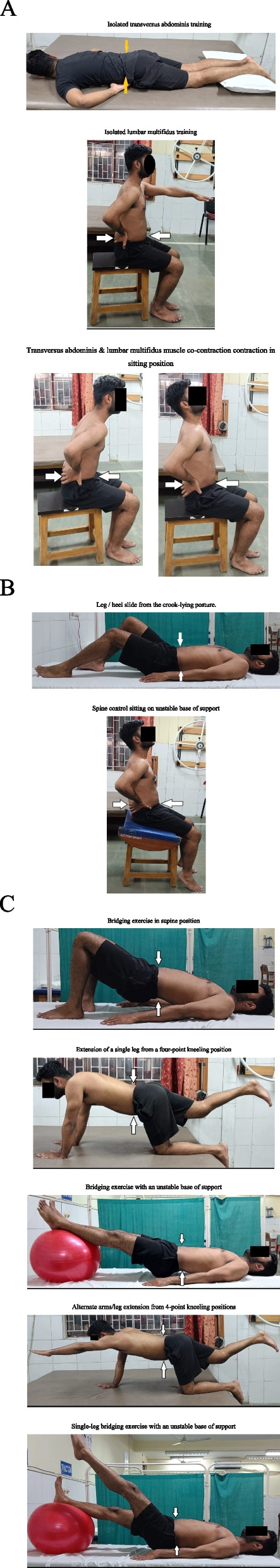
**A** Isolated transversus abdominis and lumbar multifidus training. **B** Progression of training of core muscles—lumbar stabilizing muscle activation in light dynamic functional tasks. **C** Progression of training of core muscles—lumbar stabilizing muscle activation in heavy dynamic functional tasks

Group 2 (MCE group): The first stage (1st–3rd sessions) will begin with isometric contraction of the local stability muscles (e.g., lumbar multifidus, transversus abdominis) through an abdominal drawing-in maneuver in minimally loading positions (supine lying, quadruped, sitting, and standing) [33]. The participants will be trained to activate the local stability muscles from the global muscles in an individualized manner [34]. In the second stage (4th–9th sessions), additional loads will be placed on the spine through various upper and lower extremities and trunk movement patterns to recruit various trunk [local and global] muscles. In the third stage [10th–12th sessions], functional movement patterns will be incorporated into the training program [33] (Fig. 2A–C). Exercise progression will be based on patient’s fatigue, pain thresholds, or observed movement control.

**Fig. 3:**
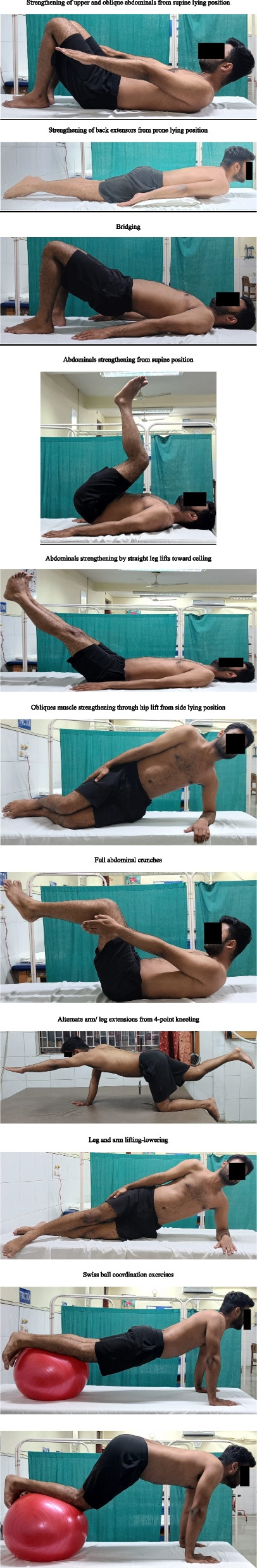
Abdominal and spine extensor muscle strengthening training

Group 3 (SS group): In this group, exercises will be focused on strengthening the rectus abdominis (RA), abdominus obliquus internus (OI), abdominus obliquus externus (OE), and erector spinae (ES) [35]. Each of the exercises will include 3 sets of 15 repetitions lasting for 45–60 min per session. Each exercise will include eight levels of steadily increasing difficulty, as outlined by Koumantakis et al. (2005) [36] (Fig. 3). Exercise progression will be based on the participant’s correct performance of the previous exercise stage and the principles of graded exposure exercise [37]. If the participants were unable to advance, they would continue exercising at the same intensity level.

Group 4 (patient education group): Health education sessions will be conducted for these participants twice a week for 12 weeks. Patient education aims to increase patients’ understanding of CLBP and alleviate myths about this condition, reassure them of the ailment’s good prognosis, and offer them useful methods for self-managing their LBP and preventing recurrence and healthcare dependency. The lecture will be followed by a 10-min discussion [38].

After the application of the study intervention, the participants will be instructed to continue the exercise interventions provided at home, twice a week, and to avoid immobility. They will be asked to report their participation using a home exercise diary.

#### Criteria for discontinuing or modifying allocated interventions {11b}

Any changes to the protocol that may affect the conduct of the study, or the potential benefit or safety of the patient, including changes to the study’s objectives, design, patient population, sample sizes, or significant administrative aspects, will necessitate a formal amendment to the protocol after agreement from the Institutional Review Boards of the Integral University and Composite Regional Centre for Skill Development, Rehabilitation, and Empowerment of Persons with Disabilities in accordance with local regulations.

#### Strategies to improve adherence to interventions {11c}

All participants will be periodically followed up over telephone. Face-to-face adherence reminder will be provided during therapy sessions or outcome measurement. The participants will be reminded about the following:

- Importance of adhering to study guidelines.
- Instructions about interventions, dosages, and the procedure to be followed in case of a missed session.
- Instructions about assessment and the procedure to be followed in case of a missed session.
- Instructions about adverse events expected, if any.
- Instructions about management of adverse events.
- Telephonic follow-up will be conducted on days 3 and 5 if the patient fails to show up for the scheduled assessment and intervention sessions.
- Importance of continuing exercises after the study period and maintaining a treatment diary.

Reasons for missing the sessions will be recorded, and strategies for incorporating exercises into everyday activities will be emphasized. From the time of recruitment, the participants will be encouraged to ask any questions, and their queries will be satisfactorily answered.

#### Relevant concomitant care permitted or prohibited during the trial {11d}

Any type of concomitant or confounding care and interventions that may influence the study outcomes will not be permitted. However, massage will be administered to the participants who develop muscle soreness after exercises. The participants will be treated with compressive garments if they complain of perceived fatigue after exercise interventions [39]. If the participants are anticipated to need an analgesic or other physiotherapy interventions during the intervention phase, they will be considered ineligible and excluded from the study.

#### Provisions for post-trial care {30}

If this study demonstrates the efficacy of any one type of exercise intervention post-treatment and at follow-up with a significant effect size, all participants will be prescribed that intervention to maximize its acceptability and use.

#### Outcomes {12}

The primary efficacy endpoint will be a minimum difference of 10% change in PPT values in CPT and a difference of ≥ 20% from baseline to week 12 in the average daily score on an 11-point (0–10) NPRS. Secondary endpoints include change from baseline to weeks 12 and 24 in number sequencing subtest, Stroop neurophysiological test, isometric muscle strength of spine and hip muscles, and the WHOQOL-BREF and ODI questionnaires.

All outcome measures will be administered or translated into Hindi for those who have difficulties reading, comprehending, and understanding English. All translations will conform to the standard guidelines for translation of patient-reported outcome measures [40].

### Primary outcome measure

#### CPM

The CPT will be used to measure CPM. For the conditioning stimulus, the participants’ non-dominant hand will be immersed in a bucket with temperature-controlled cold water (1 °C–4 °C), monitored by a thermometer for up to 1 min. The participant has to remain with the hand immersed in water without making muscle contractions or changes in position. Withdrawal from the water will be allowed if the patient can no longer tolerate the painful stimulus. This test will be conducted in a room with controlled room temperature, humidity, lighting, and noise.

Using a digital pressure algometer, the pressure pain threshold (PPT) will be performed in the forearm regions and tibialis anterior muscle of the dominant limbs before and after 1 min of the CPT. The tibialis anterior muscle and the distal part of the dorsal forearm, which had not been immersed in water, will be evaluated because of the lack of their relationship with the participants’ musculoskeletal complaints.

The operation of the pressure algometer and PPT measurement will be explained to the patients, and a practice test will be conducted on the patients’ dominant forearm to ensure that the test has been understood. Force will gradually be increased [1 kg-force/s] until the feeling of pressure changes to pain. The PPT will be recorded in kilograms force [Kgf]. The PPT will be repeated three times and the mean will be calculated to give the baseline PPT. The efficiency of the descending nociceptive inhibitory system will be evaluated by calculating the difference between PPT values in CPT (final cost − initial value) [41].

### Secondary outcomes

Executive functions will be evaluated using the letter–number sequencing subtest and the Stroop neurophysiological test.

#### Letter–number sequencing subtest

The letter–number sequencing subtest in WAIS-III will be used to evaluate working memory. In this task, the participants need to listen to a series of alphanumeric characters and repeat the characters back verbally in a specific order (numbers first in the ascending order, followed by letters in the alphabetical order). The test items will be presented in the same order for all participants. The researcher will read the test items aloud, at approximately 1 s per character. The participants will be asked to record their responses by speaking aloud to the researcher, and the researcher will mark the test stimuli as completely correct or incorrect. This subtest involves 21 items. The outcome is expressed as a scaled score; each correct response shall receive 1 point, and the maximum possible score is 21 [42].

#### Stroop neurophysiological test

The test stimuli will be presented on a computer screen. A total of 40 items will be presented in 8 rows of 5 items each. The items and the print colors will appear in a random order. The first subtask will show color words in a random order (red, blue, yellow, and green) printed in black ink. The second subtask will display solid color patches in one of these four basic colors. In the third subtask, the color words will be printed in an incongruous ink color. For example, the word *yellow* will be printed in red ink. The participants will be allowed to identify the colors in English/Hindi. No time limit will be assigned to complete a subtask. The time required in seconds to complete each Stroop subtask will be recorded using a stop clock (Stroop I, Stroop II, and Stroop III) [43]. The interference score is determined by subtracting the mean score of the first and second subtest from the amount of time required to complete the third subtest.

#### NPRS

The NPRS is a basic instrument that may be administered even to low-literate adults through a verbal face-to-face interview or telephonic interview; the adults can also self-report their responses [44]. The amount of pain felt by a patient will be subjectively estimated by marking their pain level on an 11-point, 0-to-10 NPRS, with 0 and 10 corresponding to no pain intensity and maximum pain intensity, respectively [45].

#### Oswestry Disability Index

The ODI is a 10-item questionnaire used for assessing function in the activities of daily living for LBP patients. Each item in the ODI receives a score between 0 and 5. The final score will be transformed into a percentage score. We will use the modified version of the ODI where the section on employment/homemaking ability will replace the section on sex life. This modified version has a high reliability (ICC = 0.90) and responsiveness [46]. The Hindi version of the ODI is a valid and reliable instrument for measuring disability in LBP patients [47].

### Quality of life

The participants’ QoL will be assessed using the abbreviated WHOQOL-BREF questionnaire. This questionnaire comprises 24 items divided across the 4 domains of physical health, psychological health, social relationships, and environmental health. Each item is rated on a 1–5 Likert scale. The mean score of items within each domain is summed up, and the scores are transformed on a scale from 0 to 100 [48]. The Hindi version of the WHOQOL questionnaire is effective for evaluating QoL in India’s healthcare settings [49].

### Isometric muscle strength (spine extensors, gluteus maximus, gluteus medius, and hip lateral rotators)

Isometric muscle strength of trunk extensors, transverse abdominis, obliques, gluteus medius, gluteus maximus, and lateral hip rotators will be assessed using a pressure biofeedback unit (PBU) (Chattanooga INC). Once the PBU is inflated to 70 mm Hg, the participants will be instructed to contract the specific muscles. Readings will be taken at the completion of a 10-s contraction. Two submaximal force tests will be conducted to help the participants become familiarize with each test position, followed by two repetitions with maximum isometric contraction for each muscle group. The average of the two maximum contractions will be used for analysis [50].

- *Transversus abdominis* will be tested in the prone position. The inflated cuff will be positioned beneath the abdomen at the level of the anterior superior iliac spine. The patient will be instructed to draw-in the lower abdomen without moving the upper stomach, back, or pelvis [50].
- Abdominal obliques [bilateral OE and OI] and back extensors [ES and multifidus] will be tested in the supine lying position. The inflated cell will be placed at the level of the posterior superior iliac spine.
- *Abdominal obliques*. The participants will be instructed to gently press the cell, as if to straighten the spine. For back extensors, the participants will be instructed to lift the weight off the cell [51].
- The *gluteus medius muscle* will be evaluated in the side-lying position with the test leg upward. The hip will be placed in neutral adduction and extension [52], and the inflated cuff will be placed 5 cm above the lateral condyle of the femur. The participants will be asked to lift the leg against the resistance of the examiner’s hand.
- The *gluteus maximus muscle* will be evaluated in the prone position with the hip in neutral and knee flexed to 90 degrees [53]. The inflated cuff will be placed 5 cm above the popliteal region and will attempt to lift the leg against the resistance of examiner’s hand.
- The *hip lateral rotator muscles* will be assessed in the high sitting position [54]. The inflated cuff will be placed 5 cm above the medial malleolus. The patients will be instructed to externally rotate the hip against a resistance in an opposite direction.

### Participant timeline {13}

The participant timeline is presented in Table 3.

**Table 3:** Summary of data collection

	**Enrolment**	**Baseline/assessment**	**Follow-up**	**Exit**
Subject demographics	X	X	X	X
Outcome measures	X	X	X	X
Medical history	X			
Assessment details	X	X	X	
Patient status	X	X	X	X
Events	X	X	X	X
Protocol deviations	X	X	X	X

### Sample size {14}

The sample size was calculated based on the inhibitory effect of the CPT on the PPT, assuming a standard deviation of 100 kPa for the inhibitory effect across all three exercise groups. A sample size of 30 participants per group provides 87% power for an independent *t*-test at a 2-sided alpha of 0.05 to detect a difference in inhibition of 80 kPa [17] when assuming a minimum difference of 0.47 (equivalent to 10% change) as clinically relevant for the CPM effect [55]. Therefore, anticipating a 30% dropout rate, 43 participants will be recruited for each group.

### Recruitment {15}

Various clinic- and community-based recruitment strategies will be employed to facilitate patient recruitment for the trial. Clinic-based recruitment strategies include referrals from clinicians and healthcare professionals in the study centers, as well as direct phone calls and mailings to patients from OPD records. All clinicians who agree to support the recruitment process will be provided with the detailed inclusion and exclusion criteria. The patients who agree to be contacted will be sent reminder e-mails mentioning the study requirements. Community-based recruitment strategies include web and print advertisements, flyers, and word-of-mouth referral. Advertisements will be run on Facebook and other social media platforms. Identical information regarding the inclusion criteria, estimated duration of assessment and intervention, and study contact details, including anticipated benefits, will be described in all mailings, advertisements, and flyers. All people who approach voluntarily for study participation will be inquired from where they heard about the study.

### Assignment of interventions: allocation

#### Sequence generation {16a}

Random number tables will be used to assign treatments.

#### Concealment mechanism {16b}

A sequentially numbered and sealed envelope will contain details of each successive allocation to a random treatment group. An experienced neutral researcher will be provided with sealed envelopes with randomization numbers imprinted; the randomization numbers to which the intervention groups are assigned will be revealed when the envelopes are opened.

#### Implementation {16c}

An experienced researcher not involved in patient recruitment, providing interventions, evaluating the outcomes, or following up with any participant will perform randomization.

### Assignment of interventions: blinding

#### Who will be blinded {17a}

The present study is designed as a randomized controlled trial. The intervention groups will be blinded from one another and outcome assessors. All outcome measures will be determined by an independent researcher blinded to treatment allocation. The assessor will attend training sessions on assessment procedures where the proposed assessment methods will be discussed in detail. The participants will be instructed to not disclose their intervention allocation to the outcome assessor. They will be informed that the effect of different active exercise programs will be evaluated so that they are unaware of any expected treatment group benefit. Another experienced researcher will feed the collected data into the computer. The researcher conducting the statistical analysis will not be present when the measurements are taken. All physiotherapists providing interventions will be blinded to the results of evaluations, measurements, and questionnaires.

#### Procedure for unblinding if needed {17b}

Complete blinding of investigators and participants will be attempted. The actual allocation will not be disclosed to members outside the research team. Unmasking will be permitted only under special circumstances in which knowledge of the allocated treatment is necessary for further patient care. The primary investigator will take decisions regarding unblinding in consultation with a physician.

### Data collection and management

#### Plans for assessment and collection of outcomes {18a}

All participants will be allotted unique study identifiers independent of any external source data. Using paper forms, data will be initially collected during one-on-one sessions with the participants. All data will be collected in a face-to-face interaction and stored in a secured location. The research team will collaboratively manage the data, and a statistical analyst unaware of the grouping situation or intervention measures will analyze the data.

#### Plans to promote participant retention and complete follow-up {18b}

To promote retention, feedback on measured outcomes will be provided and patient education and self-management materials regarding CLBP will be distributed. In addition, all participants will be thanked for their participation and reminded of their progress in the study, upcoming data collection, and contribution to the study results. The protocol will take a flexible approach to the study schedule, and every effort will be made to resolve any conflicts that may arise. Moreover, family support will be sought, and participants will be taught to incorporate treatment into their daily life.

#### Data management {19, 27}

Participants’ personal information and confidentiality will be protected during all trial phases. All participant data including all reports, data collection, process, and administrative forms will be housed in lockable file cabinets in restricted-access zones and shall be identifiable solely by a coded ID [identification] number. The participant’s unique number identifier, and not their names, will be displayed in all the data. Forms, lists, logbooks, appointment books, and any other listings that link participant ID numbers to other identifying information will be kept in a closed, separate file in a restricted area. Only the primary investigator will know the name associated with the number. All data collected will be converted into the electronic format and will be stored in password-protected files in a secure place at the main study centers from where the data were collected. The data will be accessible to only the research team. All personal data will be stored separately from other data and treated as confidential, will be accessible to only selected research team members, and will not be shared. The data will be retained for 5 years after study completion. Without the participant’s explicit consent, the participant’s study information will not be disclosed outside of the study.

### Statistical methods

#### Statistical methods for primary and secondary outcomes {20a,b}

The Shapiro–Wilk test will be used to assess the normality of the distribution of the data. A one-way analysis of variance (ANOVA) will be used to compare baseline characteristics between the groups. Descriptive statistics will be used to calculate the mean values of all outcome variables collected. A repeated measure ANOVA statistic will be used to evaluate the effect of three types of exercise interventions on CPM, neurophysiological outcomes, pain, disability, strength measures, and QoL. The study will use a 5 (between subjects – type of exercise interventions: AE vs. MCE vs. SS vs. patient education vs. no-pain control) × 3 (within subjects – time: before vs. after vs. 3 months after the intervention) mixed design. Tukey’s post hoc analysis will be conducted to establish differences between the groups. Effect size will be estimated by dividing the mean difference in outcomes between the intervention and control groups by the combined standard deviation of the two groups [56].

The primary outcome will undergo a per-protocol analysis. Pearson correlation analysis will be performed to determine the relationship between patient characteristics (Table 4), CPM, executive functioning, pain, strength measures, disability, and QoL. Multiple linear regression analysis will be used to deduce the characteristics of individuals responding positively to exercise interventions. For all measurements, *p* will be considered significant, if it is < 0.05. The data collected will be analyzed using SPSS (Statistical Program for the Social Sciences) 16.0 (IBM Corp., Armonk, NY).

**Table 4:** Summary of patient characteristics to be collected

Characteristics	CLBP group	Control group
Age (years)	X	X
Gender (male/female)	X	X
Pain episode duration (weeks)	X	X
Body mass index (kg/m^2^)	X	X
Education level (yes/no; school incomplete, completed secondary school, completed diploma/completed bachelors and above)	X	X
Employment status (yes/no; employed (full or part-time), unemployed, retired)	X	X
Marital status (yes/no; married/separated/ divorced, single)	X	X
Personal behaviors Alcohol status (yes/no) Smoking status (yes/no)	X	X
Comorbidities	X	X
Fear avoidance behavior	X	X
Perception of improvement	X	X

The results will be considered clinically significant if a minimum difference of 10% change in PPT values in CPT is maintained at 6 months. Based on a previous study results [57] and a standardized mean score of 10 [58] on the letter–number sequencing subtask of WAIS-III, the mean reaction time for the Stroop interference effect will be fixed at approximately 230 s for the treatment to be considered successful. A reduction of 2 points on the NPRS [59], a cut-off value of 13, and a difference of 8 and 4 points will be considered as the minimal clinically important difference for the ODI [60], WHOQOL-BREF [61], and isometric muscle strength [62] respectively, which would indicate the success of the therapeutic intervention used.

#### Methods for handling protocol non-adherence and statistical methods for handling missing data {20c}

The research team will undertake all measures to minimize missing of data, and reasons for missing data, if any, will be ascertained using sensitivity analyses. Likelihood-based methods will be used if these reasons are rational. If the data are found to not miss at random, selection models and pattern-mixture models will be used. The *intent-to-treat* analysis will be used to evaluate our objectives based on the groups to which the participants are originally assigned after data transformation was performed.

#### Adverse event reporting and harms {22}

Therapeutic safety will be monitored on the basis of patient symptoms. Changes in pain, CPM, muscle soreness, and lumbar function degradation will be meticulously documented in the case report form.

#### Frequency and plans for auditing trial conduct {23}

We intend to conduct an audit every 6 weeks to examine all parts of research processes including participant enrolment, consent, eligibility, and allocation to study groups, as well as adherence to trial interventions, reporting of harms, accuracy, and ensuring timeliness of data collection.

#### Dissemination plans {31a}

The study results will be published in peer-reviewed indexed journals and distributed to the participants, healthcare professionals, the general public, and other relevant parties.

#### Publication {31b}

The results will be published irrespective of the outcome. Authorship shall be determined in accordance with the International Committee of Medical Journal Editors’ criteria. The authors have no limits on publication.

## Discussion

Although physical exercises have been considered as the foundation of management of musculoskeletal pain conditions with proven benefits [63], the precise mechanisms underlying the effects remain unclear [64]. Traditional explanations based on biomechanics and corresponding changes in loading of the musculoskeletal system regarding why exercise improves pain and disability in chronic musculoskeletal pain fail to consider the complete biopsychosocial spectrum of factors [65]. Exercise prescription is usually based on the preferences and expertise of the individual clinician. Because exercise prescription directly impacts intervention outcomes, analyzing the exercise parameters in patients along the chronic pain spectrum is crucial.

The study will offer the following benefits. First, it will help in acquiring more information and evidence about exercise-induced analgesia from the CPM perspective. Second, investigating the relationship between executive functions, CPM, and QoL will provide further inputs for high-quality randomized controlled trials in the future. Third, measuring the outcomes of exercises will help in scientifically prescribing different exercise types. The study outcomes will also help in identifying the characteristics of individuals responding or responding indifferently to exercises; this will avoid wastage of health resources or assist in directing the non-responsive individuals to other alternative interventions.

### Study strengths

The study intends to investigate only those outcome measures that have good psychometric properties, have been used extensively in research, and can be measured quickly. We will only recruit participants aged between 18 and 59 years as a previous study has hinted at a decline in endogenous pain modulatory and CPM functions with advancing age [66]. Studying the association between executive functioning, CPM, and exercises will be a major addition to the results as a previous study has indicated that this relationship is selectively sensitive and the positive effect observed on animals do not translate in humans [67]. Further, we will apply the optimal exercise dose in each group based on the methodology used in previous works.

### Study limitations

Limiting the study to CS-positive participants will limit the generalization of the results. Long-term effects of exercises on CPM will not be evaluated, and the results may not be applicable to the older population. The role of confounding factors such as gender, body mass index, and level of physical activities will not be considered in this study. Future studies should evaluate the role of potential confounders associated with the CPM response.

## Trial status

Protocol version number: 3 (October 15, 2022).

First day of recruitment: October 17, 2022.

Expected end of recruitment: January 17, 2023.

## Supplementary Information

**Additional  file 1.**

## Data Availability

The datasets used and/or analyzed during the current study will be available from the corresponding author on reasonable request.

## Abbreviations

AE: Aerobic exercise
ANOVA: Analysis of variance
CLBP: Chronic low back pain
CNS: Central nervous system
CONSORT: Consolidated Standards of Reporting Trials
CPM: Conditioned pain modulation
CPT: Cold pressor test
CS: Central sensitization
EIH: Exercise-induced hypoalgesia
ES: Erector spinae
LBP: Low back pain
MCE: Motor control exercise
NPRS: Numerical Pain Rating Scale
NSCLBP: Non-specific chronic low back pain
ODI: Oswestry Disability Index
OE: Obliquus externus
OI: Obliquus internus
PBU: Pressure biofeedback
PPT: Pressure pain threshold
QoL: Quality of life
RA: Rectus abdominis
SD: Standard deviation
SPIRIT: Standard Protocol Items: Recommendations for Interventional Trials
SPSS: Statistical Program for the Social Sciences
SS: Superficial strengthening
TrA: Transversus abdominis
