# Publisher Correction: Effectiveness of motor control exercise, aerobic walking, and muscle strengthening programs in improving outcomes in a subgroup of population with chronic low back pain positive for central sensitization: a study protocol for a randomized controlled trial

**DOI:** 10.1186/s13063-023-07370-5

**Published:** 2023-06-01

**Authors:** G. Shankar Ganesh, Abdur Raheem Khan, Sakti Prasad Das, Ashfaque Khan, Raee S. Alqhtani, Adel Alshahrani, Mohammad Abdulrehman Mohammad Jarrar, Hashim Ahmed

**Affiliations:** 1 Composite Regional Centre for Skill Development, Rehabilitation, and Empowerment of Persons with Disabilities, Lucknow, Uttar Pradesh 226017 India; 2 Integral University, Kursi Road, Lucknow, Uttar Pradesh 226026 India; 3 Swami Vivekanand National Institute of Rehabilitation Training and Research, Cuttack Dt, Odisha, 754010 India; 4 College of Applied Medical Sciences, Najran University, Najran, 55461 Kingdom of Saudi Arabia

**Publisher Correction: Trials 24, 319 (2023)**

**https://doi.org/10.1186/s13063-023-07316-x**

The original publication of this article [1] contained a duplicated author name. The correct author list is available in this correction article, the original article has been updated.
