# Supplementary material for: Effectiveness of motor control exercise, aerobic walking, and muscle strengthening programs in improving outcomes in a subgroup of population with chronic low back pain positive for central sensitization: a study protocol for a randomized controlled trial
Source: Trials. 2023 May 9;24:319. doi: 10.1186/s13063-023-07316-x (PMC10169487; doi:10.1186/s13063-023-07316-x)
Supplement: Supplementary file 1 — Additional file 1. [file 13063_2023_7316_MOESM1_ESM.docx]

**Informed Consent forms** for men and women who are aged between 18 and 59 years of age who have CLBP and are positive for central sensitizaion-related signs and symptoms will be recruited from the Outpatient Physiotherapy Department of the Composite Regional Centre for

Skill Development, Rehabilitation, and Empowerment of Persons with Disabilities. Similarly,

we will recruit a no-pain control population within the age group and who we are invited to participate in the research . The title of our research project is "Effectiveness of motor control exercise, aerobic walking, and muscle strengthening programs in improving outcomes in a subgroup of population with chronic low back pain positive for central sensitization: a study protocol for a randomized controlled trial"

**[Name of Principal Investigator]: G. Shankar Ganesh**

**[Name of Organization]:** Composite Regional Centre for Skill Development, Rehabilitation, and Empowerment of Persons with Disabilities**,** Lucknow

**[Name of Co-investigator]: Dr. Abdur Raheem Khan**

**[Name of Organization]: Integral University, Lucknow**

**This Informed Consent Form has two parts:**

- **Information Sheet (to share information about the research with you)**
- **Certificate of Consent (for signatures if you agree to take part)**

**You will be given a copy of the full Informed Consent Form**

**PART I: Information Sheet**

**Introduction**

I am G.Shankar Ganesh, working for the Composite Regional Centre for Skill Development, Rehabilitation, and Empowerment of Persons with Disabilities. We are doing research to evaluate if different types of exercises studied can improve outcomes in a sub-group of patients with chronic low back pain (CLBP) positive for central sensitization. I am going to give you information and invite you to be part of this research. You do not have to decide today whether or not you will participate in the research. Before you decide, you can talk to anyone you feel comfortable with about the research.

There may be some words that you do not understand. Please ask me to stop as we go through the information and I will take time to explain. If you have questions later, you can ask them of me or the staff.

**Purpose of the research**

"Chronic low back pain" (CLBP) refers to low back pain that is persistent and incapacitating for longer than three months in nature and is unrelated to underlying illnesses like an infection, tumour, or fracture. Studies have indicated abnormal cortical function in people with CLBP such as disruption of executive functions like multi-tasking ability, sustained attention and working memory, longer processing time and significant disturbances in mental flexibility, delayed memory, and psychomotor speed.

The role of increased pain sensitivity in the development and maintenance of chronic pain states and their influence on patient recovery is known as central sensitization (CS). Conditioned pain modulation (CPM) is widely used to measure musculoskeletal pain associated with CS. CPM is found to be directly related to cognitive performance and self-reported health-related quality of life (QoL). It is believed that persons suffering from chronic pain have lower inhibition of CPM and exercises are believed to reduce the overall sensitivity of the central nervous system. However, past reviews and guidelines have hardly provided little detail about the indications of different types of exercise to be used in clinical practice and no previous works have studied the associations between CPM outcomes, executive functions and quality of life [QoL] in persons with CLBP from the perspective of exercise interventions. Thus, more studies on the effects of varied exercise types and dosage on exercise induced hypoalgesia in chronic pain populations that more closely resemble clinical practice are required. The reason we are doing this research is to find out if there is a change in CPM values in patients with CLBP and study the effect of 3-different types of exercise interventions in a sub-group of patients positive for CS. We also intend to explore the relationship between CPM responses, CLBP related outcomes, and executive functions in this population.

**Data to be collected**

This research will collect the following information based on your performance in the following standardized tests on 3 different timeframes:

1. **CPM** – using the Cold Pressor Test [CPT]
2. **Executive functions** will be evaluated using the Letter-Number Sequencing subtest and the Stroop neurophysiological test.
3. **Pain intensity** will be measured by numerical pain rating scale (NPRS)
4. **The disability** associated with low back pain will be measured by Oswestry Disability Index (ODI*)*
5. **Quality of Life (*QoL)*** will be assessed by the abbreviated WHOQOL-BREF
6. **Isometric Muscle Strength (spine extensors, gluteus maximus, gluteus medius, and hip lateral rotators)** will be assessed using a pressure bio-feedback (PBU) unit

**Participant selection**

We are inviting all cognitively intact adults aged between 18-59 years of age suffering from CLBP of more than 3 months duration, having pain intensity of more than 4 on the NPRS, and

positive for CS-related sign and symptoms to participate in the research to evaluate if 3-types of active exercise interventions modify the outcomes.

**Voluntary Participation**

Your participation in this research is entirely voluntary. It is your choice whether to participate or not. Whether you choose to participate or not, all the services you receive at this department, currently or in the future will continue and nothing will change. If you choose not to participate in this research project, you will be offered the treatment that is routinely offered in this department for CLBP, and we will tell you more about it later. You may change your mind later and stop participating even if you agreed earlier.

**Procedures and Protocol**

Because we do not know if there is any relationship exercise prescription in improving the outcomes in persons suffering from CLBP, we need to determine if different types of exercises produce varied outputs on CPM in a sub-group of patients with CLBP classified as having CS pain. Further, there is a need to evaluate if a program of exercise interventions for 12 weeks would alter executive functioning, QoL, disability. To do this, we will put people taking part in this research into 3-different types of exercises.

The participants who will participate in this intervention will not know what exercise interventions other participants are prescribed. It is important that the researchers who evaluate the outcomes do not know about this information. This information will be in our files, but we will not look at these data files until after the research is finished. This is the best way we have for testing without being influenced by what we think or hope might happen. We will then measure the outcomes and identify the relationship between the collected variables. If there is anything you are concerned about or that is bothering you about the research please talk to me or one of the other researchers.

You will undergo the interventions and tests to evaluate the outcomes according to established procedures. This means that you will be expected to undergo interventions up to 12-weeks and outcome tests on up to 3-different timeframes. One investigator will be with you you at all times during the exercise and measurement of outcomes.

During the research, participants will undertake:

- 3-different active exercise interventions along with patient education and 1-passive intervention comprising of patient education alone for 12-weeks (lasting 30-60 minutes).
- One clinical test each will measure the intensity of pain, disability meted out by CLBP, and the QoL based on a standard questionnaire.
- Two tests (Letter-Number Sequencing subtest in the Wechsler Adult Intelligence Scale and Stroop Neurophysiological Test) to measure the brain executive functions. You will be asked to listen to a series of alphanumeric characters and repeat the characters back verbally in a specific order. Another test will present **t**he test stimuli on a computer screen. You will be asked to identify the colours in 3-different scenarios.
- One test will measure the isometric strength of your spine muscles.
- One test will measure the CPM. You will be required to keep your hand in cold water, while the investigator will measure the pressure pain threshold [PPT] from 2-different body regions.

**Duration**

The research takes place over 6 months in total. During that time, it will be necessary for you to come to the department/hospital for 12 weeks for exercises. Follow-up will occur 3 and 6 months after the intervention. At the end of six months, the research will be finished.

**Side Effects**

As already mentioned, you will be required to undergo certain clinical tests and exercises. These active exercises can make you tired and it is possible that it may also cause some problems that we are not aware of. However, we will follow you closely and keep track of any unwanted effects or any problems. If this is necessary we will discuss it together with you and you will always be consulted before we move to the next step.

**Risks**

No specific adverse events related to the study are expected for patients. Common adverse events associated with this kind of treatments are those injuries which might occur following exercises, at the out-patient unit, or related to transport. While the possibility of this happening is very low, you should still be aware of the possibility. We will try to decrease the chances of this event occurring, but if something unexpected happens, you would be managed in accordance with current rules of good clinical practice. All adverse events will be reported to the regulatory authority and the Ethics Committee in accordance with relevant regulations.

**Benefits**

The study provides a direct benefit for each patient in that all patients will receive standardized physiotherapy treatment that is consistent with evidence based recommendations. Future studies may study the effectiveness of treating the variables studied and future generations are likely to benefit.

The outcomes of the study will also result in the following benefits: first, it will help in gaining more information and evidence about exercise-induced analgesia from the perspective of CPM. Secondly, studying the relationship between executive functions, CPM and QoL will provide further inputs for high-quality RCTs in the future. Thirdly, by measuring the outcomes to exercises will help in scientifically prescribing different types of exercises. The study outcomes will also assist in identifying the characteristics of individuals who will respond or respond indifferently to exercises; this will avoid wasting of health resources or assist in directing those persons to other alternative interventions.

**Reimbursements**

You will not be given any monetary benefits or gifts to take part in this research.

**Confidentiality**

We will not be sharing the identity of those participating in the research. The information that we collect from this research project will be kept confidential. Information about you that will be collected during the research will be put away and no-one but the researchers will be able to see it. Any information about you will have a number on it instead of your name. Only the researchers will know what your number is and we will lock that information up with a lock and key. It will not be shared with or given to anyone.

**Sharing the Results**

The knowledge that we get from doing this research will be shared with you before it is made widely available to the public. Confidential information will not be shared. There may be small meetings in the institute or amongst the research team. After these meetings, we will publish the results in order that other interested people may learn from our research.

**Right to Refuse or Withdraw**

You do not have to take part in this research if you do not wish to do so. You may also stop participating in the research at any time you choose. It is your choice and all of your rights will still be respected.

**Who to Contact**

If you have any questions you may ask them now or later, even after the study has started. If you wish to ask questions later, you may contact any of the following:

1. G. Shankar Ganesh, 9437279869, [shankarpt@rediffmail.com](mailto:shankarpt@rediffmail.com)
2. Dr.Abdur Raheem Khan, 9616739965, abdurraheem@iul.ac.in

This study has been approved by the institute ethics committee, Integral University (IIAHSR/DO/PT/2022/23) and Composite Regional Centre for Skill Development, Rehabilitation, and Empowerment of Persons with Disabilities (CRCL/Ph.D.Data Collection/2021-22/1556) and registered under access code CTRI/2022/03/041143 in the Clinical Trial Registry of India (CTRI).

**PART II: Certificate of Consent**

**I have read the foregoing information, or it has been read to me. I have had the opportunity to ask questions about it and any questions that I have asked have been answered to my satisfaction. I consent voluntarily to participate as a participant in this research.**

**Print Name of Participant__________________**

**Signature of Participant ___________________**

**Date ___________________________**

**Day/month/year**

**If illiterate**

**I have witnessed the accurate reading of the consent form to the potential participant, and the individual has had the opportunity to ask questions. I confirm that the individual has given consent freely.**

**Print name of witness_____________________ AND Thumb print of participant**

**Signature of witness ______________________**

**Date ________________________**

**Day/month/year**

**Statement by the researcher/person taking consent**

**I have accurately read out the information sheet to the potential participant, and to the best of my ability made sure that the participant understands that the following will be done:**

**1.**

**2.**

**3.**

**I confirm that the participant was given an opportunity to ask questions about the study, and all the questions asked by the participant have been answered correctly and to the best of my ability. I confirm that the individual has not been coerced into giving consent, and the consent has been given freely and voluntarily.**

**A copy of this ICF has been provided to the participant.**

**Print Name of Researcher****/person taking the consent________________________**

**Signature of Researcher /person taking the consent__________________________**

**Date ___________________________**

**Day/month/year**
